# Porous Copolymers of 1,4-Di(methacryloxymethyl)naphthalene (DMN) with Trimethylpropane Trimethacrylate (TRIM)—Synthesis, Characterization, and Post-Crosslinking Modification

**DOI:** 10.3390/ma19153161

**Published:** 2026-07-23

**Authors:** Małgorzata Maciejewska, Barbara Gawdzik

**Affiliations:** Department of Polymer Chemistry, Institute of Chemical Sciences, Faculty of Chemistry, Maria Curie-Skłodowska University, Gliniana 33, 20-614 Lublin, Poland

**Keywords:** porous microspheres, aromatic methacrylates, thermal stability, gas chromatography stationary phase

## Abstract

Porous microspheres based on 1,4-(dimethacryloyloxymethyl)naphthalene (DMN) and trimethylolpropane trimethacrylate (TRIM) were obtained by suspension–emulsion polymerization in the presence of toluene as a porogenic diluent. The obtained copolymers were subsequently modified using tetrachloromethane in the presence of anhydrous AlCl_3_ via a Friedel–Crafts-type reaction. The influence of monomer composition and post-polymerization modification on the porous structure parameters and thermal stability of the materials was investigated. The synthesized copolymers exhibited well-developed porous structures with surface areas ranging from 368 to 494 m^2^/g. Increasing the TRIM content resulted in higher crosslinking density, earlier phase separation during polymerization, and formation of a finer porous architecture characterized by increased surface area and lower pore diameters. Chemical modification caused moderate and composition-dependent changes in the porous structure while preserving the mesoporous character of the materials. The highly crosslinked copolymers demonstrated the greatest structural stability during modification. Thermogravimetric analysis performed in helium revealed high thermal resistance of both parent and modified copolymers. The degradation process proceeded in two main stages characteristic of highly crosslinked methacrylate networks. Increasing TRIM content improved resistance toward advanced thermal decomposition, increasing the T_50%_ values up to 415 °C. Post-polymerization modification slightly decreased the temperature of the second degradation stage, probably due to the introduction of thermally less stable chlorinated fragments, while simultaneously increasing char residue formation. The synthesized materials were also evaluated as stationary phases for gas chromatography. Owing to their high thermal stability and the presence of polar ester functionalities, the copolymers enabled efficient separation of aliphatic alcohols at elevated temperatures. The obtained results demonstrate that porous poly(DMN-co-TRIM) microspheres constitute promising thermally stable materials with tunable porous structure and potential applications in chromatographic separation techniques.

## 1. Introduction

Highly crosslinked porous polymers constitute an important class of materials due to their well-developed internal structure, high physicochemical stability, and wide range of practical applications [1,2,3,4]. Such materials have attracted considerable attention as adsorbents, catalyst supports, separation media, ion-exchange materials, and stationary phases in chromatographic techniques [2,3,4,5,6,7,8,9,10]. Their applicability results mainly from the possibility of tailoring porous structure, surface chemistry, and thermal resistance through appropriate selection of monomers and polymerization conditions. Among the various classes of porous polymeric materials, crosslinked methacrylate-based copolymers are particularly attractive because they combine good mechanical properties, chemical resistance, and relatively simple synthesis procedures [11,12,13,14,15,16,17,18].

The porous structure of highly crosslinked copolymers is governed by the polymerization conditions, especially the nature of the porogenic solvent and the degree of crosslinking of the polymer network. Under suspension polymerization conditions, in the presence of an inert diluent, phase separation occurs at a certain stage of conversion, leading to the formation of polymer-rich and solvent-rich domains. Upon removal of the porogenic solvent, a permanent porous structure is generated. The final textural properties, such as specific surface area, pore volume, and pore size distribution, depend primarily on the kinetics of phase separation and the rigidity of the growing polymer network. Therefore, the introduction of multifunctional monomers capable of forming highly crosslinked structures is an effective strategy for obtaining porous materials with high thermal and structural stability.

Particular interest has recently been devoted to porous polymers containing aromatic moieties within the polymer backbone. Aromatic units increase the rigidity of the network and may significantly improve thermal resistance. Moreover, aromatic rings enable further post-polymerization functionalization through electrophilic substitution reactions, providing an additional route for tuning the physicochemical properties of the materials. In this context, methacrylate monomers containing condensed aromatic systems appear especially promising because they combine the reactivity of methacrylate groups with the advantageous properties of rigid aromatic structures [19,20,21,22,23,24,25,26,27].

Previously, porous copolymers based on 9,10-bis(methacryloyloxymethyl)anthracene and trimethylolpropane trimethacrylate (TRIM) were reported to exhibit a highly developed porous structure and very good thermal stability [28]. The favorable thermal properties observed for the anthracene-based system provided the rationale for investigating analogous naphthalene-based systems. In the present study, aromatic 1,4-(dimethacryloyloxymethyl)naphthalene (DMN) was synthesized and subsequently copolymerized with TRIM under modified suspension polymerization conditions in the presence of a porogenic solvent (toluene). The incorporation of naphthalene moieties into the polymer framework was expected to provide rigid aromatic domains contributing to improved thermal resistance and favorable adsorption properties. An additional objective of the study was to chemically modify the synthesized porous copolymers using tetrachloromethane in the presence of anhydrous AlCl_3_. Friedel–Crafts-type reactions performed on aromatic polymer networks may alter the surface chemistry and porous structure of the materials, thereby influencing their thermal behavior and potential application properties. Therefore, both the parent and modified copolymers were comprehensively characterized with respect to their porous structure and thermal stability.

Special attention was also devoted to the evaluation of the applicability of the synthesized materials as stationary phases for gas chromatography. Owing to their high thermal resistance, developed porous structure, and the presence of polar ester groups capable of interacting with polar analytes, the obtained copolymers appear to be promising materials for chromatographic separations performed at elevated temperatures.

**Figure 1:**
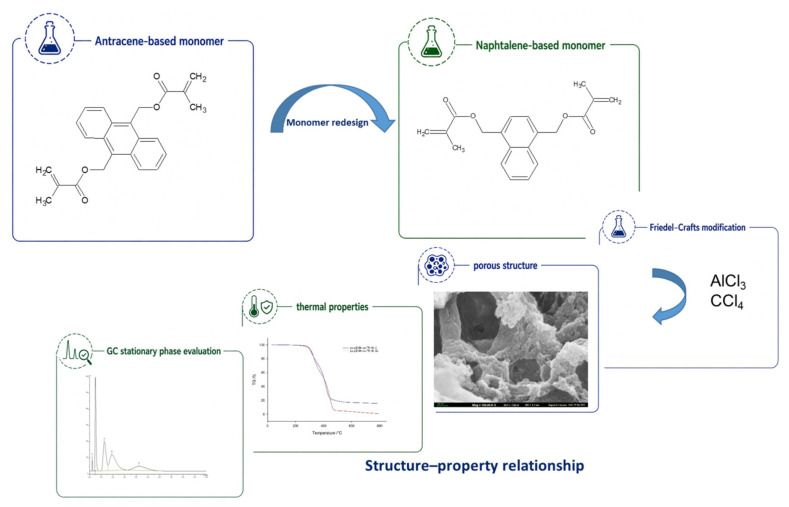
Graphical overview of the present study illustrating the transition from the previously investigated anthracene-based copolymer to the newly developed naphthalene-based system, followed by Friedel–Crafts modification and comprehensive characterization of the resulting porous microspheres.

Accordingly, the present study was undertaken to investigate how the DMN/TRIM molar ratio and subsequent Friedel–Crafts post-polymerization modification affect the porous architecture, thermal stability, and chromatographic performance of the synthesized porous microspheres (Figure 1).

## 2. Materials and Methods

### 2.1. Chemicals

TRIM was obtained from Sigma–Aldrich (Schnelldorf, Germany) and washed with 5% aqueous sodium hydroxide to remove inhibitors. 1,4-(dimethacryloyloxymethyl) naphthalene (DMN) was synthesized in the Department of Polymer Chemistry (UMCS, Lublin, Poland) in accordance with the method described elsewhere [29]. 2,2′-Azoisobutyronitrile (AIBN) and bis(2-ethylhexyl) sulfosuccinate sodium salt (AOT) from Fluka AG (Buchs, Switzerland) were used without purification. 1,2-dichloroetane, acetone, toluene, benzene, hexan-1-ol, pentan-1-ol, butan-1-ol, propan-1-ol ethanol, methanol, and sodium hydroxide were from POCh (Gliwice, Poland).

### 2.2. Synthesis of Permanently Porous Microspheres

The continuous aqueous phase was prepared by dissolving 2.2 g of dioctyl sulfosuccinate sodium salt (AOT) in 195 mL of deionized water. Separately, the organic phase was prepared by dissolving 15 g of the monomer mixture (DMN and TRIM) together with 0.2 g of azobisisobutyronitrile (AIBN) in 22.5 mL of toluene. The organic phase was then gradually introduced into the aqueous phase under continuous stirring. The molar ratio of DMN to TRIM was varied from 1:1 to 1:5 in order to investigate the influence of copolymer composition on the properties of the resulting materials. Polymerization was carried out at 80 °C for 20 h. The resulting porous microspheres were isolated by filtration using 5 μm filter paper.

**Figure 2:**
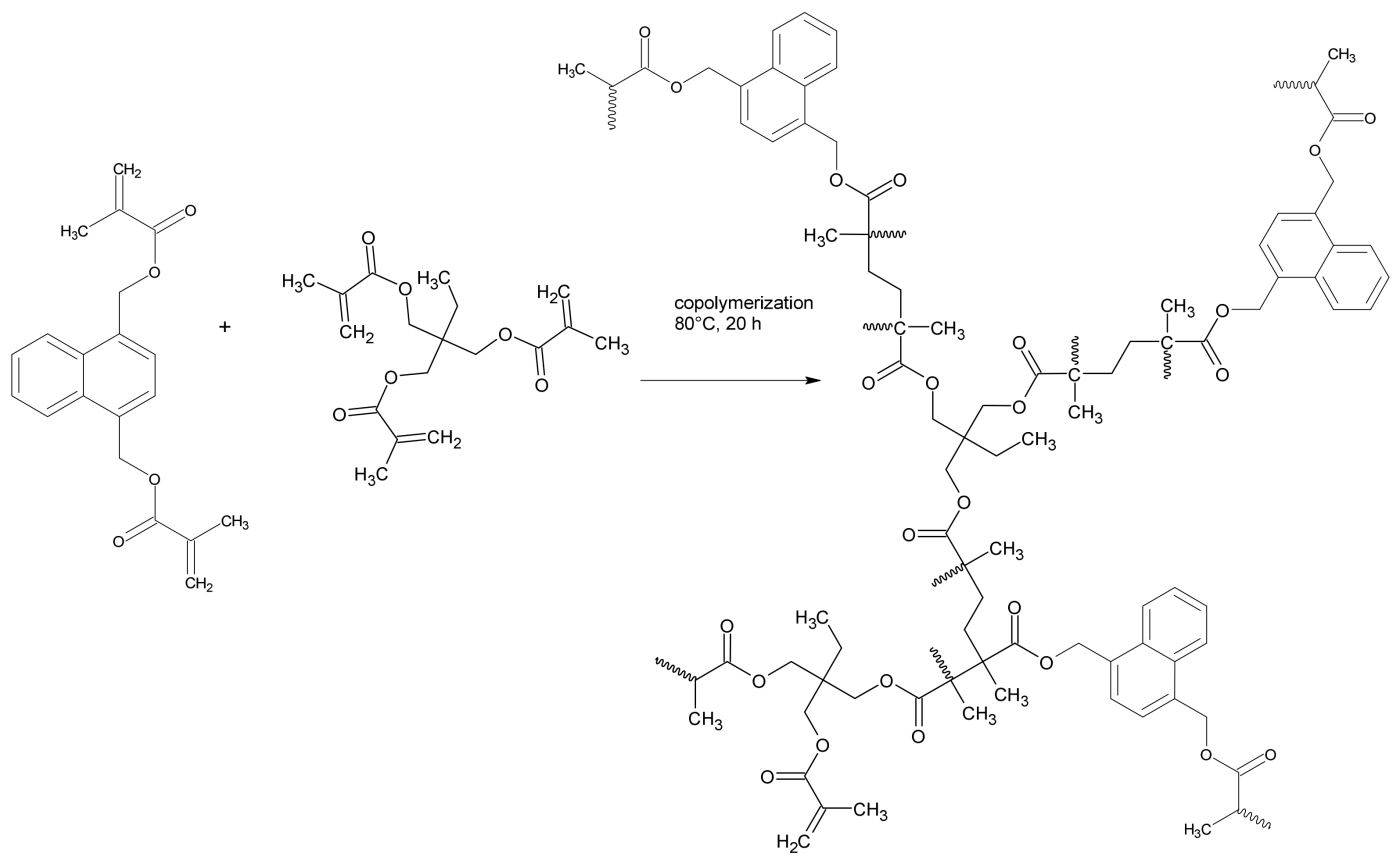
Schematic representation of the suspension–emulsion copolymerization of DMN with TRIM. Adapted from Ref. [30].

To remove residual monomers, porogenic diluent, and physically adsorbed surfactant, the copolymer microspheres were subjected to a multistep purification procedure. The beads were first dispersed in deionized water and sonicated for 30 min. After decantation of the aqueous phase, they were resuspended in methanol and sonicated for an additional 30 min. Subsequently, the microspheres were transferred to a round-bottom flask, suspended in toluene, and stirred for 30 min. The toluene was then removed, and the microspheres were stirred in methanol for a further 30 min. Finally, the methanol was decanted, the microspheres were thoroughly washed with deionized water, filtered, and dried under reduced pressure at 80 °C for 48 h. A schematic representation of the synthesis procedure is shown in Figure 2.

*2,3 Post-Crosslinking Modification*

The porous copolymers were chemically modified via a Friedel–Crafts reaction. Initially, 1.28 g of anhydrous AlCl_3_ was mixed with 2 mL of freshly distilled tetrachloromethane and cooled to 0–5 °C. After stirring for 1 h, the reaction mixture was allowed to warm to 10–15 °C, and 2 g of porous copolymer, previously swollen in 15 mL of 1,2-dichloroethane for 25 h, was added. The reaction mixture was then cooled to −20 °C under continuous stirring and maintained at this temperature for 4 h. Subsequently, the temperature was raised to 15 °C, and the reaction was continued for an additional 24 h. Upon completion of the reaction, the modified copolymer was isolated by filtration and washed successively with 1,2-dichloroethane, ethanol, and distilled water to remove residual reagents and catalyst. Finally, the product was thoroughly rinsed with distilled water and dried under reduced pressure (1.3 kPa) at 80 °C.

### 2.3. Measurements Methods

Fourier transform infrared (FTIR) spectra were recorded using a Tensor 27 FTIR spectrometer (Bruker Corp., Ettlingen, Germany) equipped with an attenuated total reflectance (ATR) accessory. The spectra were collected over the range of 4000–600 cm^−1^ at a spectral resolution of 4 cm^−1^, averaging 32 scans for each measurement.

The porous structure of the copolymers was characterized by nitrogen adsorption–desorption measurements performed at −196 °C using an ASAP 2405N analyzer (Micromeritics Corp., Norcross, GA, USA). Prior to analysis, the samples were degassed under vacuum (10^−2^ mm Hg) at 80 °C overnight. The specific surface area (S_BET_) was calculated according to the Brunauer–Emmett–Teller (BET) method, whereas the Barrett–Joyner–Halenda (BJH) approach was employed to determine the total pore volume (V), pore size distribution (PSD), and the corresponding pore diameter (D_BJH_), estimated from the maximum of the pore size distribution curve.

The morphology of the synthesized microspheres was examined using a Quanta 3D FEG DualBeam™ scanning electron microscope (FEI Corp., Hillsboro, OR, USA) operating at an accelerating voltage of 5 kV. Prior to imaging, the samples were sputter-coated with a thin layer of gold to improve their electrical conductivity.

Surface topography was further investigated using a Contour GT optical profilometer (Bruker Corp., Ettlingen, Germany).

The swellability of the copolymers was evaluated by equilibrium swelling experiments carried out in methanol, toluene, acetone, and 1,2-dichloroethane using the centrifugation method. The swelling coefficient (B) was calculated according to Equation (1):

(1) $$B=\frac{V_{s}-V_{d}}{V_{s}}\times 100\%$$

where *V_s_* is the volume of the copolymer after swelling, and *V_d_* is the volume of the dry copolymer.

Differential scanning calorimetry (DSC) measurements were performed using a DSC 204 calorimeter (Netzsch, Selb, Germany) operating in dynamic mode. Samples weighing 10.0 ± 0.2 mg were placed in pierced aluminum pans (approximately 40 mg), while an empty aluminum pan served as the reference. Measurements were carried out from room temperature to 500 °C under a flow of argon (30 mL min^−1^) at a heating rate of 10 °C min^−1^.

Thermogravimetric analyses (TG) were conducted using a Netzsch STA 449 F1 Jupiter thermal analyzer (Netzsch, Selb, Germany). The measurements were performed over the temperature range of 35–700 °C under either helium or synthetic air flowing at 20 mL min^−1^, employing a heating rate of 10 °C min^−1^. Approximately 10.0 ± 0.2 mg of sample was placed in Al_2_O_3_ crucibles (approximately 160 mg), while an empty crucible was used as the reference. To eliminate physically adsorbed moisture, all samples were preheated at 100 °C prior to the measurements.

The volatile products evolved during the thermal decomposition of selected copolymers were identified by coupling the thermogravimetric analyzer online with a Bruker Tensor 27 FTIR spectrometer (Bruker, Hanau, Germany). The instruments were connected via a 2 mm diameter PTFE transfer line maintained at 200 °C to prevent condensation of volatile decomposition products. Gas-phase FTIR spectra were collected in the 600–4000 cm^−1^ spectral range at a resolution of 4 cm^−1^, averaging 16 scans per spectrum.

The chromatographic performance of the synthesized copolymers was evaluated using a Dani GC 1000 gas chromatograph (Dani, Milan, Italy) equipped with a packed-column injector maintained at 220 °C and a thermal conductivity detector (TCD) operated at the same temperature. Helium was used as the carrier gas at a flow rate of 50 mL min^−1^. Samples were manually injected using a 1 μL microsyringe (SGE, North Melbourne, Australia). Chromatographic separations were performed at 140 °C and 200 °C.

## 3. Results

**Figure 3:**
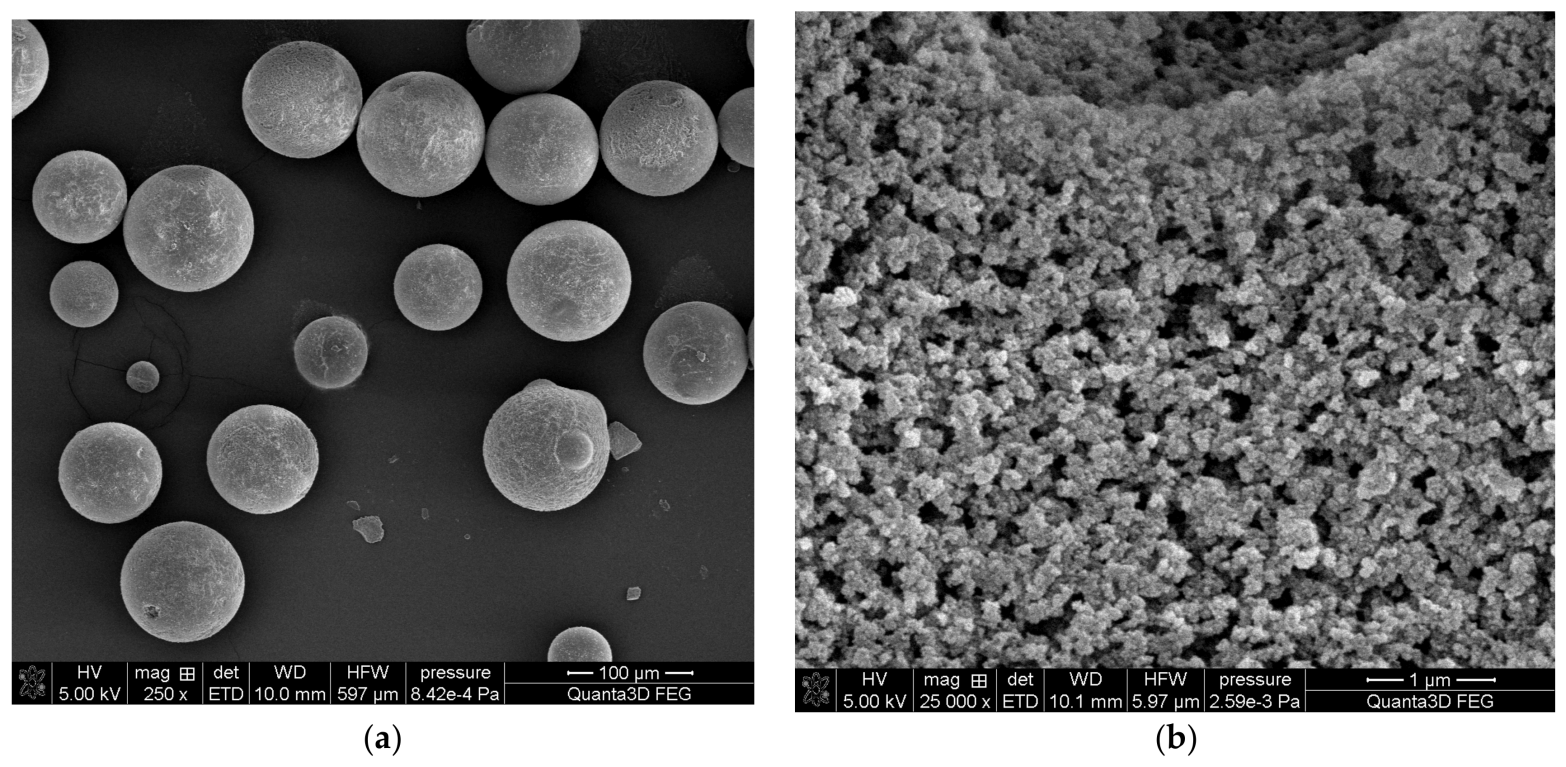
SEM micrographs of the poly(DMN-co-TRIM)_5 copolymer showing (**a**) the overall morphology of the microspheres (250×) and (**b**) the surface morphology at magnification (25,000×).

Porous poly(DMN-co-TRIM) copolymers were synthesized using the suspension–emulsion polymerization technique. This method, extensively developed and optimized in our department, combines the most advantageous features of both classical suspension and emulsion polymerization processes. In comparison with conventional suspension polymerization, the applied approach enables the formulation of more uniform particles and a well-developed porous structure. At the same time, unlike typical emulsion polymerization, it allows the preparation of larger polymer beads that can be easily isolated and handled in further physicochemical investigations and practical applications. A characteristic feature of the applied method is the use of an oil-soluble radical initiator (AIBN) instead of a conventional water-soluble initiator typically employed in emulsion systems. As a result, polymerization occurs predominantly within the dispersed organic droplets containing monomers, porogenic solvent, and initiator. This modification of the polymerization system significantly affects the particle formation and growth, leading to the generation of spherical polymeric microspheres with diameters of 25–80 µm (Figure 3).

The obtained microspheres exhibit good spherical morphology and a relatively narrow particle-size distribution, which are highly advantageous for applications involving adsorption and chromatographic separation processes. Moreover, the applied suspension–emulsion technique facilitates effective heat transfer during polymerization and enables efficient control of the porous structure formation through adjustment of the composition of the organic phase and polymerization conditions. Consequently, this method constitutes an effective route for the preparation of thermally stable porous microspheres with tailored physicochemical properties.

**Figure 4:**
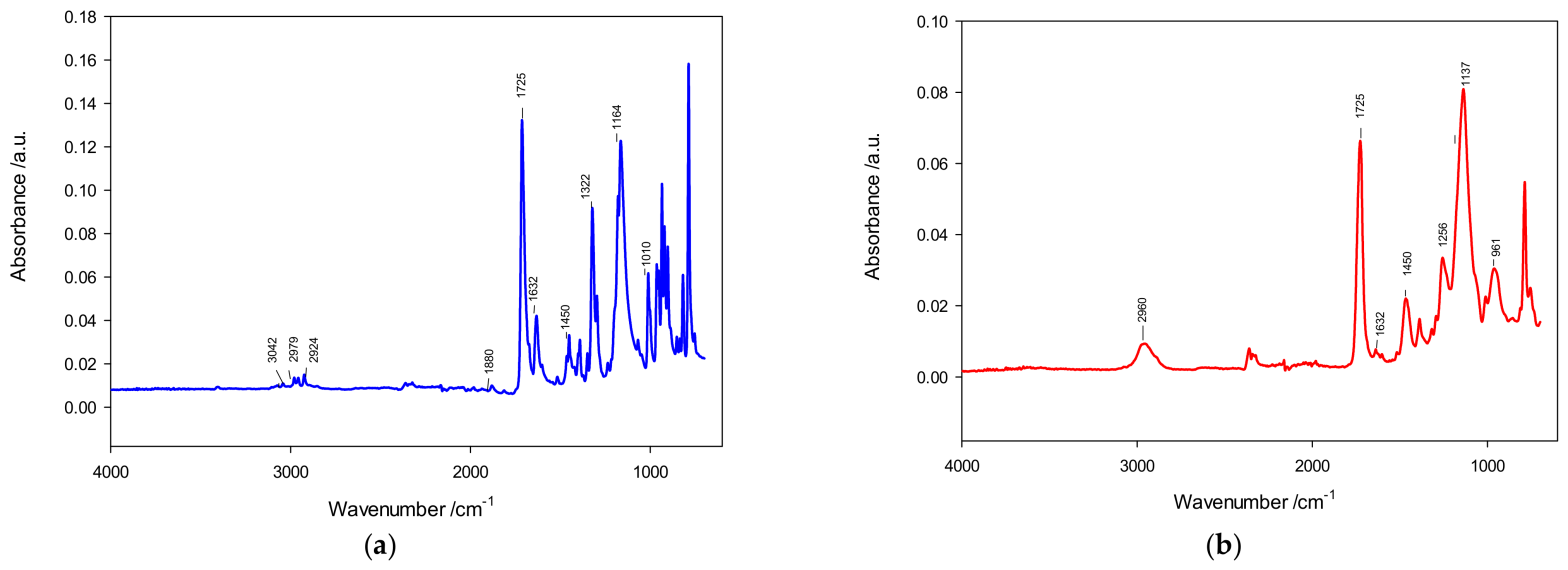
ATR spectra of the DMN monomer, blue line (**a**) and poly(DMN-co-TRIM) copolymer, red line (**b**).

The polymerization process was monitored using infrared spectroscopy. Figure 4 presents FTIR-ATR spectra of the DMN monomer (a) and its copolymer (b).

As can be seen in the DMN spectrum (a), the bands characteristic of alkyl and alkylene groups, i.e., at 2979–2924 cm^−1^ (ν C–H) and 1465–1450 cm^−1^ (δ C–H), are visible. The presence of the ester group is revealed by the band at 1725 cm^−1^ (ν C=O) and confirmed by bands at 1164–1010 cm^−1^ (ν C–O). The unsaturated double bonds are represented by the band at 1632 cm^−1^ (ν C=C) and confirmed by overtone at 1880 cm^−1^, a small peak at 3042 cm^−1^ (ν =C–H), and multiple peaks at 920–902 cm^−1^ (γ =C–H). Moreover, the 1,4-disubstituted naphthalene ring system matches well with the band at 818 cm^−1^ (δ C-H out-of-plane). After polymerization, peaks connected with unsaturated double bonds (1632 cm^−1^, 3042 cm^−1^) considerably diminished their intensity (Figure 4b).

The use of toluene as a porogenic diluent during polymerization resulted in the formation of highly porous copolymeric microspheres. The polymerization was carried out for a series of monomer feed ratios ranging from DMN:TRIM = 1:1 to 1:5. As the proportion of TRIM in the reaction system increased, the obtained copolymers exhibited a progressively more developed porous structure. Consequently, the specific surface area increased from 368 m^2^/g for poly(DMN-co-TRIM)_1 to 494 m^2^/g for poly(DMN-co-TRIM)_5 (Table 1). Simultaneously, the pore size distribution shifted toward smaller pore diameters.

The observed structural evolution can be explained by the mechanism of phase separation occurring during the formation of crosslinked polymer networks. At the onset of suspension polymerization, droplets of the organic phase are dispersed within the aqueous medium under stirring conditions, forming a homogeneous solution containing monomers, initiator, and porogenic solvent. As polymerization proceeds and the degree of conversion increases, the initially homogeneous organic phase becomes thermodynamically unstable and separates into polymer-rich and solvent-rich domains. The polymer-rich phase forms the rigid crosslinked network, whereas the solvent-rich phase ultimately generates the porous structure after removal of the diluent.

The phase separation process responsible for pore formation is generally attributed either to an increase in crosslinking density (ν-syneresis) or to changes in interactions of polymer and solvent (χ-syneresis). Since both the type and amount of porogenic solvent were kept constant throughout the experiments, the differences in porous structure can primarily be related to variations in the crosslinking degree resulting from the changing monomer composition. TRIM, containing three methacrylate groups per molecule, exhibits significantly higher crosslinking capability than the difunctional aromatic monomer DMN. Therefore, increasing the TRIM content in the polymerization mixture enhances the overall crosslinking density of the forming polymer network. In highly crosslinked systems, phase separation occurs at earlier stages of polymerization, resulting in the nucleation of numerous small primary polymer particles.

Owing to their rigid and densely crosslinked structure, these nuclei possess limited swelling ability and reduced capacity for monomer absorption. As a consequence, the concentration of unreacted monomers inside the nuclei remains relatively low, which substantially decreases the predisposition of adjacent nuclei to coalesce. Consequently, the polymerization system retains a high population of small, discrete microscale domains, generating a porous network composed predominantly of smaller pores. This effect is reflected in the BJH pore diameter data, where samples containing larger amounts of TRIM exhibit lower average pore diameters together with higher specific surface areas. The increase in surface area therefore originates not only from the higher number of pores formed, but also from the finer porous architecture produced by early-stage phase separation and restricted coalescence phenomena.

The results demonstrate that the porous structure of poly(DMN-co-TRIM) microspheres can be effectively tailored through adjustment of the monomer composition, particularly by controlling the content of the highly crosslinking TRIM monomer. The observations are consistent with the general behavior reported for highly crosslinked porous organic polymers, where enhanced crosslinking promotes earlier phase separation and the formation of a more developed porous network. Similar relationships between crosslinking density, pore development, and surface area have been reported for hypercrosslinked polymer systems [31,32].

Based on the previously conducted investigation of the influence of Friedel–Crafts reaction on the properties of poly(styrene-co-divinylbenzene) porous copolymers [33,34], the obtained microspheres were swollen in 1,2-dichlorethane and treated with tetrachloromethane. Anhydrous AlCl_3_ acted as a catalyst. The post-polymerization reaction led to measurable, but composition-dependent, changes in the porous structure (Table 2).

Since the copolymers were pre-swollen in 1,2-dichloroethane prior to modification, the reaction could proceed not only at the external surface of the microspheres but also within the accessible pore system. Therefore, the final textural properties reflect the balance between chemical functionalization of the internal surface, possible pore-wall rearrangement, partial filling or narrowing of pores, and preservation of the original crosslinked morphology. For most samples, the modified microspheres retained relatively high specific surface areas, ranging from 383 to 498 m^2^/g, and pore volumes between 0.398 and 0.535 cm^3^/g. This confirms that the crosslinked DMN–TRIM framework is sufficiently rigid to withstand Friedel–Crafts-type treatment under strongly Lewis-acidic conditions. Compared with the parent copolymers, samples poly(DMN-co-TRIM)_1c and poly(DMN-co-TRIM)_2c showed a slight increase in specific surface area, from 368 to 383 m^2^/g and from 386 to 403 m^2^/g, respectively. Their pore volumes also increased slightly. This may indicate that, in systems with lower TRIM content and therefore lower crosslinking density, swelling in 1,2-dichloroethane followed by chemical modification caused partial opening of previously less accessible pores. The polymer network in these samples was probably more flexible, allowing better penetration of the reaction medium into the internal structure. For poly(DMN-co-TRIM)_5c, the porous structure was almost fully preserved. The specific surface area remained essentially unchanged, increasing only slightly from 494 to 498 m^2^/g, while the pore volume changed only marginally from 0.450 to 0.44 7 cm^3^/g. This indicates that the highly crosslinked framework formed at the highest TRIM content provides the greatest structural stability during modification. The rigid network likely limits excessive swelling, pore collapse, and secondary structural rearrangements. The BJH pore diameter values also show that the modified copolymers remained predominantly mesoporous. In the initial samples, bimodal pore size distributions were observed for the 1:1, 1:2, and 1:3 copolymers. After modification, bimodality was retained for samples 1c and 2c, whereas sample 3c exhibited a narrower distribution centered around 4 and 6 nm. For samples 4c and 5c, the pore system remained mainly unimodal, with pore diameters close to 4 nm. These results suggest that chemical modification affects mainly the accessibility and relative contribution of particular pore populations rather than completely rebuilding the porous network. Overall, the modification with tetrachloromethane/AlCl_3_ caused moderate changes in the porous structure, but the direction and scale of these changes depended powerfully on the initial monomers’ composition. Less crosslinked materials showed slight development of accessible porosity, probably due to enhanced swelling and opening of the network. In contrast, intermediate compositions showed partial loss of surface area, most likely due to pore narrowing or blockage. The most highly crosslinked material, poly(DMN-co-TRIM)_5c, exhibited excellent textural stability, maintaining the highest surface area after modification.

The swelling experiments provide additional insight into the network architecture of the synthesized copolymers (Table 3). Swelling of highly crosslinked polymers is governed by two competing factors, namely the accessibility of the porous structure and the elasticity of the polymer network. Consequently, the highest swelling coefficients were observed for poly(DMN-co-TRIM)_3, suggesting that this composition offers the most favorable balance between pore accessibility and network rigidity. Further increasing the TRIM content led to lower swelling despite the higher specific surface area, indicating that the increasing crosslinking density progressively limits the expansion of the polymer network. After Friedel–Crafts modification, the swelling coefficients decreased markedly for all copolymers, confirming that the post-polymerization reaction increased the effective rigidity of the network while preserving its porous character.

Compared with styrene–divinylbenzene copolymers [33,34], the poly(DMN-co-TRIM) microspheres exhibited a more complex and composition-dependent response toward Friedel–Crafts-type modification. This difference may be attributed to the distinct chemical nature and rigidity of the polymer networks. The styrene–divinylbenzene matrix, although highly crosslinked, contains relatively flexible aliphatic–aromatic segments and phenyl rings that are readily accessible to electrophilic substitution reactions. Consequently, post-crosslinking often results in the formation of additional bridges within the polymer framework, leading to a substantial increase in specific surface area and pore volume. For example, Zeng et al. [35] reported a significant development of porous structure after post-crosslinking of poly(divinylbenzene) (PDHT-1), attributing the increase in specific surface area to the formation of an additional microporous network during hypercrosslinking. Consistent findings were subsequently reported by Soukupová and Jeřábek [36], who also observed a pronounced increase in the surface area of post-crosslinked poly(divinylbenzene). It should be noted, however, that the increase in the dry-state specific surface area of conventional macroreticular polymers after Friedel–Crafts post-crosslinking is generally associated with the formation of a large number of micropores. Although such a structural transformation is advantageous for adsorption processes involving small molecules, it does not necessarily improve the accessibility of the pore wall surface for larger species. Therefore, a high increase in BET surface area is not always directly correlated with improved performance in applications requiring efficient mass transport through the porous network. In contrast, the DMN–TRIM copolymers investigated in the present study already possess a highly crosslinked and rigid architecture resulting from the presence of multifunctional methacrylate groups and fused naphthalene rings. Such a structure limits the extent of network rearrangement during the Friedel–Crafts reaction and reduces the possibility of creating new microporous domains. As a result, only moderate changes in specific surface area and pore volume were observed after modification. The preservation of the original porous architecture suggests that the reaction predominantly affects the surface chemistry and local pore geometry rather than inducing extensive restructuring of the polymer network.

From an application point of view, this behavior may be advantageous because the desired chemical modification can be introduced without substantial loss of mesoporosity and pore accessibility. Consequently, the modified materials retain their favorable transport properties while simultaneously acquiring new surface functionalities.

**Figure 5:**
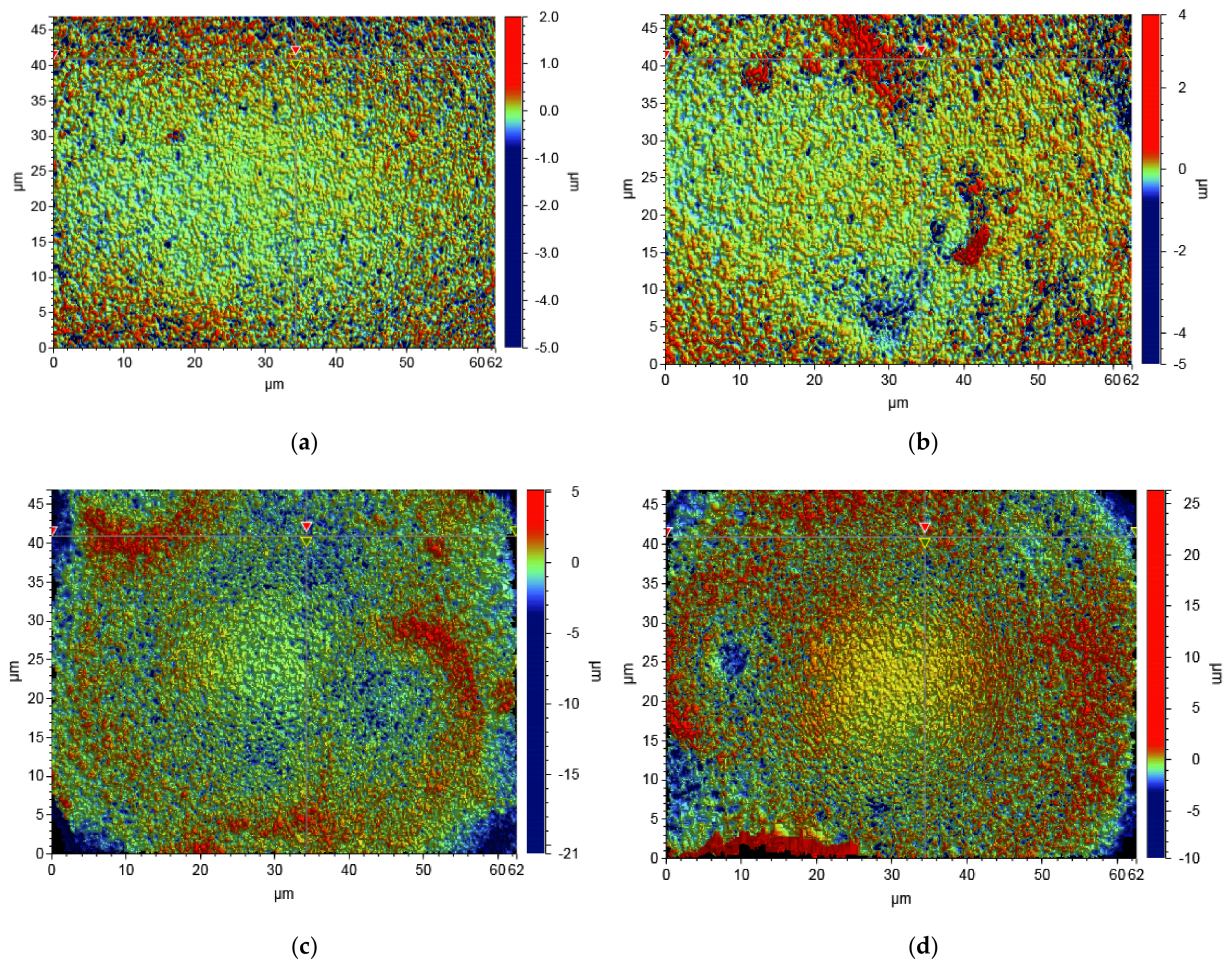
Three-dimensional surface topography of (**a**) poly(DMN-co-TRIM)_1, (**b**) poly(DMN-co-TRIM)_1c, (**c**) poly(DMN-co-TRIM)_3, and (**d**) poly(DMN-co-TRIM)_3c obtained by optical profilometry.

To gain additional insight into the influence of chemical modification on the external morphology of the microspheres, optical profilometry measurements were performed. Optical profilometry is a non-destructive surface characterization technique that provides quantitative information about the topography and roughness of a material. Representative optical profilometry images of the investigated microspheres are presented in Figure 5. The three-dimensional surface maps reveal noticeable differences in the external morphology of the copolymers before and after chemical modification. In agreement with the nitrogen adsorption results, the copolymers containing a higher proportion of TRIM exhibit a more developed surface topography than the corresponding 1:1 systems. Furthermore, the modified samples display a more heterogeneous surface characterized by a greater number of surface irregularities and height variations. To quantify these observations, the arithmetic mean roughness (Ra) and the root mean square roughness (Rq) parameters were determined. Ra describes the average deviation of the surface profile from the mean plane and is commonly used as a general indicator of surface development, whereas Rq corresponds to the root mean square roughness and is particularly sensitive to pronounced peaks and valleys; therefore, it provides additional information regarding surface heterogeneity.

The obtained results confirmed changes in the surface topography of the copolymeric microspheres after chemical modification. For poly(DMN-co-TRIM)_1, the Ra value increased from 0.209 to 0.259, while Rq increased from 0.345 to 0.406. A similar, but more pronounced, tendency was observed for poly(DMN-co-TRIM)_3, for which Ra increased from 0.957 to 1.215 and Rq from 1.705 to 1.800 after modification. The increase in both Ra and Rq parameters indicates that the Friedel–Crafts-type treatment leads to the formation of a more developed and heterogeneous surface topography. This effect may result from swelling of the polymer network in 1,2-dichloroethane, penetration of the reaction medium into accessible surface regions, and subsequent chemical modification accompanied by local structural rearrangements. The higher Rq values compared with Ra suggest the presence of more pronounced local irregularities, such as surface depressions or protrusions, rather than uniformly distributed roughness. Moreover, the substantially higher roughness values observed for poly(DMN-co-TRIM)_3 compared with poly(DMN-co-TRIM)_1 indicate that the monomer composition and crosslinking density strongly influence not only the internal porous structure but also the external morphology of the microspheres. The increase in surface roughness after modification supports the conclusion that the chemical treatment affects the surface architecture while preserving the overall porous character of the materials.

The roughness parameters are consistent with the visual observations from Figure 5 and correlate well with the textural characteristics determined from nitrogen adsorption measurements, indicating that chemical modification affects not only the internal porous structure but also the external surface architecture of the microspheres.

After evaluation of the porous structure and the impact of post-polymerization modification on the textural parameters of the synthesized microspheres, the next stage of the study focused on their thermal properties. Investigation of the thermal behavior is particularly important for highly crosslinked porous materials, since both the monomer composition and the chemical modification procedure may significantly affect the thermal stability and decomposition mechanism of the polymer network. Therefore, thermogravimetric analysis was conducted to evaluate the effect of the DMN/TRIM ratio as well as the tetrachloromethane/AlCl_3_ modification on the resistance of the materials toward thermal degradation.

**Figure 6:**
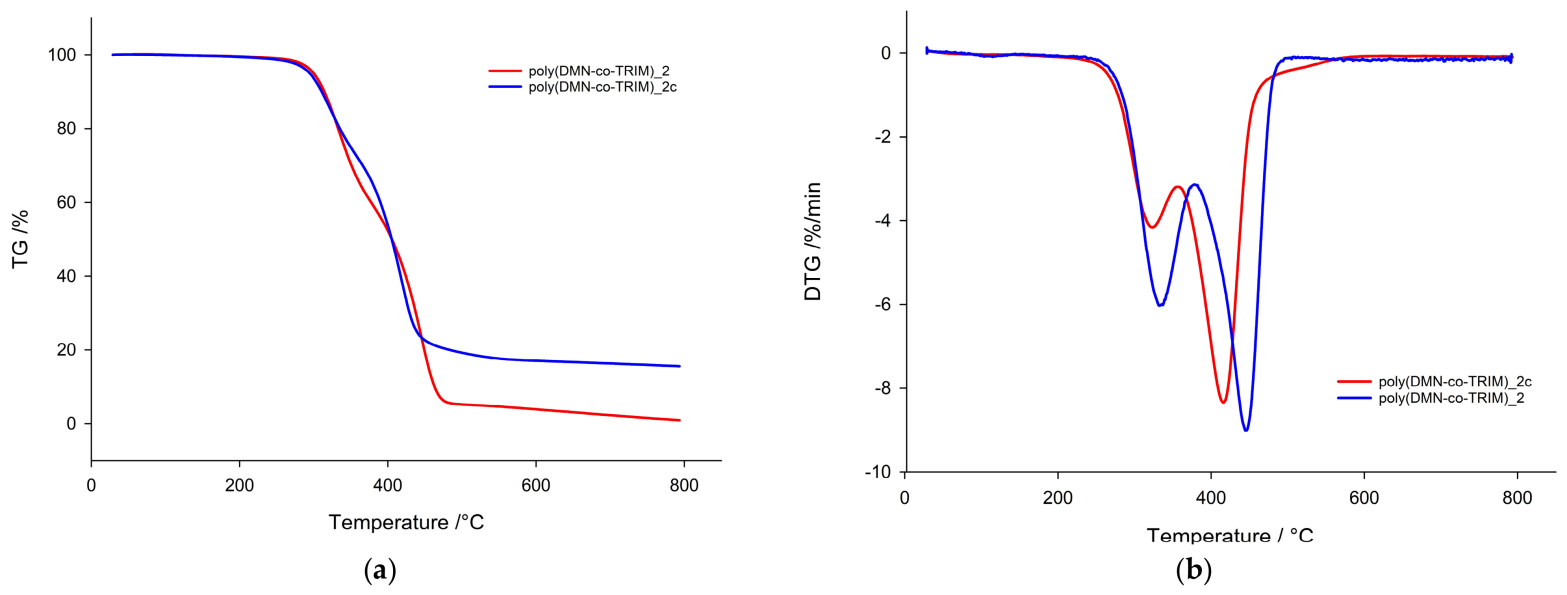
TG (**a**) and DTG (**b**) curves of the parent poly(DMN-co-TRIM)_2 and modified poly(DMN-co-TRIM)_2c copolymers determined in helium.

The TG/DTG analysis performed under helium demonstrated that all parent poly(DMN-co-TRIM) microspheres exhibit high thermal stability (Table 4, Figure 6). The temperatures corresponding to 5% mass loss (T_5%_) fall within a relatively narrow range of 292–301 °C, indicating that the onset of thermal degradation is only weakly dependent on the monomer composition. More pronounced differences between the copolymers become visible at higher conversion degrees, particularly for T_50%_ and the positions of DTG maxima.

The degradation profiles of all investigated copolymers reveal a two-step decomposition mechanism. The first degradation stage occurs at approximately 324–334 °C, while the second stage is observed at significantly higher temperatures, in the range of 445–453 °C. The first stage may be associated with the decomposition of less thermally stable fragments, including cleavage of ester groups and degradation of less densely crosslinked regions. The second stage corresponds to the decomposition of the rigid aromatic network formed by highly crosslinked TRIM-rich domains and DMN-containing structures. For poly(DMN-co-TRIM)_1, the first degradation step accounts for 45.9% mass loss, while the second stage contributes 52.6%. For the remaining copolymers, the first stage corresponds to approximately 40% mass loss and the second stage to 57–59%. Thus, increasing the TRIM content only slightly modifies the relative contributions of both degradation stages, while preserving the overall two-step decomposition pathway. The influence of the monomer composition is most clearly reflected in the T_50%_ values. As the TRIM content increases, T_50%_ rises from 400 °C for the 1:1 copolymer to 415 °C for poly(DMN-co-TRIM)_5. This trend confirms that higher amounts of trifunctional TRIM lead to the formation of a more densely crosslinked polymer network with enhanced resistance toward advanced thermal degradation. The increase of T_50%_ with increasing TRIM content is consistent with the generally accepted effect of crosslinking density on thermal stability. Similar behavior has been reported for various crosslinked methacrylate and aromatic polymer networks, where highly crosslinked structures exhibit delayed decomposition and enhanced resistance toward thermal degradation [37].

A similar tendency is visible for the second DTG maximum, which shifts from 446 °C for poly(DMN-co-TRIM)_1 to 453 °C for the copolymers with the highest TRIM content. The relatively small variation in T_5%_ values suggests that the initial degradation process is controlled mainly by the decomposition of similar ester-containing fragments present in all copolymers. In contrast, the later stages of degradation strongly depend on the degree of crosslinking and network rigidity. The presence of aromatic naphthalene rings in DMN additionally contributes to thermal resistance by increasing structural stiffness and restricting segmental mobility of the polymer chains. All copolymers leave only trace amounts of residue after degradation under helium, ranging from 0.1 to 2.9%. Such low char yields indicate that decomposition proceeds predominantly through chain scission and volatilization of degradation products rather than through carbonization processes. This behavior is typical of highly crosslinked methacrylate-based materials.

Overall, TG/DTG results confirm that the poly(DMN-co-TRIM) microspheres possess very good thermal stability. Increasing the TRIM content systematically improves resistance to advanced thermal decomposition by enhancing the crosslinking density and rigidity of the polymer network, while the general degradation mechanism remains similar for all investigated compositions. The modified poly(DMN-co-TRIM) microspheres retain high thermal stability, with T_5%_ values between 292 and 306 °C (Table 5). This indicates that the tetrachloromethane treatment does not significantly reduce the onset of degradation. The decomposition still proceeds in two main stages, as in the parent copolymers. The first DTG maximum occurs at 323–333 °C, while the second appears at 416–433 °C. The first stage can be assigned to the degradation of less stable methacrylate-derived fragments, including ester bond cleavage and decomposition of more labile network regions. The second stage is related to the degradation of the more rigid aromatic and highly crosslinked polymer structure.

Compared with the unmodified copolymers, the most important difference is the lower T_max2_ values after modification (Figure 6). For the parent materials, T_max2_ was around 445–453 °C, whereas for the modified samples it decreased to 416–433 °C. This suggests that chemical modification introduces structural elements that are less thermally stable than the original DMN–TRIM network. These may include newly introduced chlorinated or trichloromethyl-type groups formed during the Friedel–Crafts-type reaction [33,34]. Their presence can promote earlier decomposition of the aromatic network, probably through cleavage of C–Cl-containing fragments or destabilization of adjacent polymer segments. The T_50%_ values of the modified copolymers remain relatively high, 386–405 °C, confirming that the materials still possess good resistance to thermal degradation. The lowest value of T_50%_ possesses poly(DMN-*co*-TRIM)_1c copolymer. This may indicate that the lowest-crosslinked material is more susceptible to structural changes during modification. Its network is more flexible and more accessible to the reaction medium, which may result in a higher local concentration of thermally labile modified fragments.

The mass-loss distribution also changes after modification. For the modified samples, the first degradation step accounts for 33–44.5%, while the second step accounts for 45.5–57%. This confirms that the two-stage degradation mechanism is preserved, but the relative contributions of the stages are affected by chemical modification. Samples with higher TRIM content, especially poly(DMN-*co*-TRIM)_4c and poly(DMN-*co*-TRIM)_5c, show a larger contribution of the second degradation stage, consistent with the presence of a more densely crosslinked framework. A particularly important result is the substantial increase in residue after degradation. The parent copolymers left only 0.1–2.9% residue, whereas the modified copolymers left 7.3–15.5%. This indicates that the chemical modification strongly changes the degradation pathway. The introduced chlorinated aromatic structures may promote char formation and reduce the complete volatilization of the polymer.

**Figure 7:**
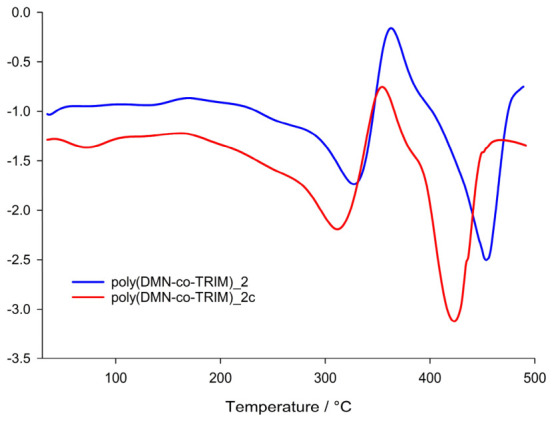
DSC curves of the parent poly(DMN-co-TRIM)_2 and modified poly(DMN-*co*-TRIM)_2c copolymers determined in argon.

In addition to thermogravimetric analysis, differential scanning calorimetry (DSC) was performed to characterize the thermal effects associated with the decomposition of the copolymers. The DSC measurements enabled the identification of endothermic and exothermic events occurring during heating and provided complementary information regarding the degradation pathways inferred from the TG/DTG results. Figure 7 shows DSC curves of the parent and modified copolymers determined in an inert atmosphere (argon).

The DSC thermograms revealed two endothermic effects at approximately 313 and 461 °C. These temperatures are significantly higher than those expected for glass transition phenomena in highly crosslinked methacrylate-based networks. Moreover, the observed thermal effects occur in the same temperature regions as the main decomposition stages determined from TG/DTG analysis. Therefore, the recorded DSC peaks should be attributed to endothermic processes accompanying thermal degradation of the polymer network rather than to polymer-chain segmental mobility or glass transition events.

Overall, the modified copolymers remain thermally stable, but their degradation behavior differs clearly from that of the parent materials. The modification does not significantly affect the initial degradation temperature, but it lowers the temperature of the second decomposition stage and strongly increases the solid residue. This means that the Friedel–Crafts-type modification introduces thermally labile groups that decompose earlier, while simultaneously enhancing char formation at high temperature. The final thermal behavior is therefore controlled by two opposite effects: partial destabilization of the main degradation stage and increased formation of non-volatile residue.

**Figure 8:**
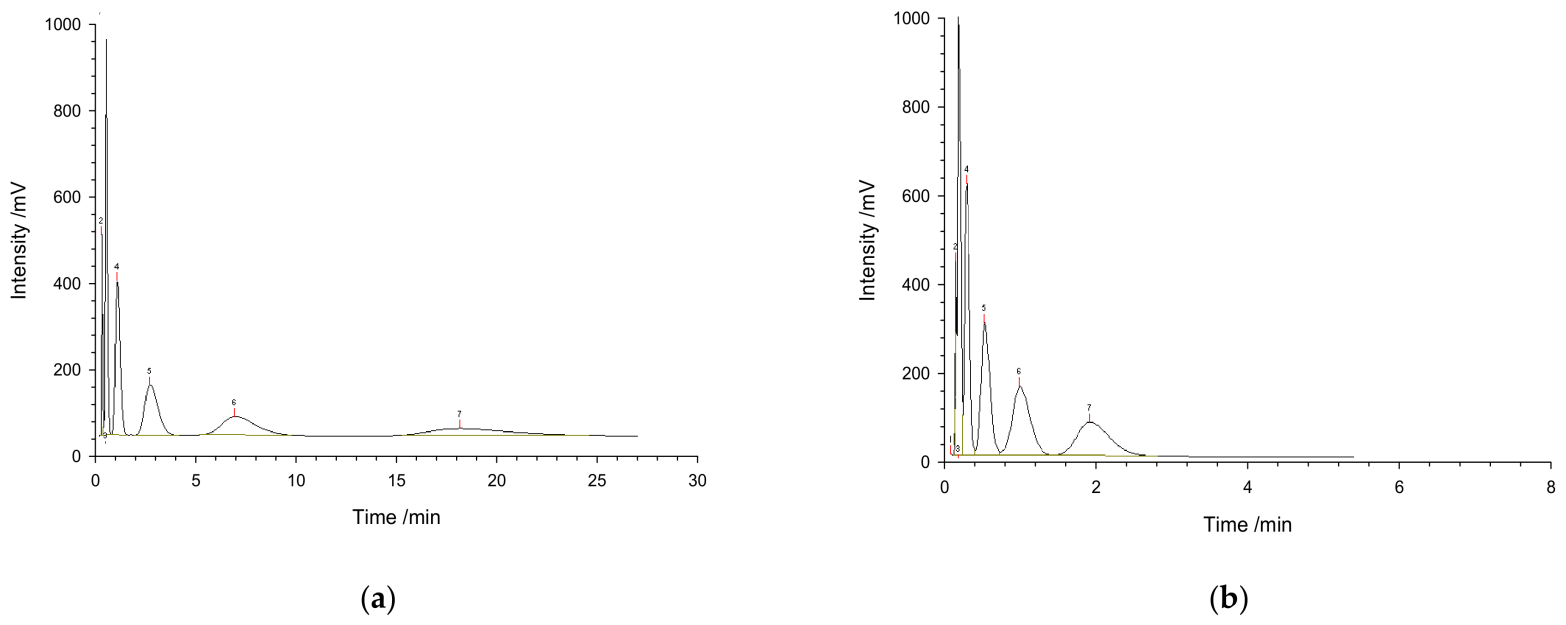
GC chromatograms demonstrating the separation of a homologous series of aliphatic alcohols on the poly(DMN-co-TRIM)_5c stationary phase at (**a**) 140 °C and (**b**) 200 °C. Peaks: 1—H_2_O, 2—CH_3_OH, 3—CH_3_CH_2_OH, 4—CH_3_CH_2_CH_2_OH, 5—CH_3_CH_2_CH_2_CH_2_OH, 6—CH_3_CH_2_CH_2_CH_2_CH_2_OH, 7—CH_3_CH_2_CH_2_CH_2_CH_2_CH_2_OH.

The high thermal stability of the synthesized poly(DMN-co-TRIM) copolymers makes them promising candidates for application as stationary phases in gas chromatography. In chromatographic systems operated at elevated temperatures, thermal resistance of the stationary phase is a crucial parameter because it determines not only the operational stability of the material but also the efficiency and reproducibility of separation processes. The obtained TG/DTG results demonstrated that the investigated copolymers retain structural integrity at temperatures significantly exceeding typical GC operating conditions, which indicates their potential suitability for chromatographic applications. An additional advantage of the synthesized materials arises from the presence of ester groups within the polymer network. These polar functionalities impart a partially hydrophilic character to the copolymers and enable specific interactions with polar analytes, particularly compounds capable of hydrogen bonding, such as alcohols. Consequently, the retention behavior of aliphatic alcohols on the investigated stationary phase is governed not only by dispersive interactions but also by dipole–dipole interactions between the hydroxyl groups of the analytes and the ester moieties present in the polymer structure. Figure 8 presents chromatograms obtained for the separation of a mixture of aliphatic alcohols on poly(DMN-co-TRIM)_5c copolymer. The chromatographic performance of the synthesized copolymer was evaluated using the same experimental approach as described in our previous study on polymeric monoliths [38]. However, the present chromatograms were obtained for a different stationary phase and a different alcohol mixture and therefore represent new experimental results.

The obtained results clearly demonstrate the strong influence of operating temperature on chromatographic performance. At 200 °C, the separation process proceeds considerably faster than at 140 °C. For example, the retention time of hexan-1-ol decreases from 18.76 min at 140 °C to only 2.51 min at 200 °C. Such behavior is associated with the increased volatility of analytes and weaker analyte–stationary phase interactions at elevated temperature, which facilitate faster mass transfer through the column.

Moreover, the chromatographic peaks recorded at 200 °C are noticeably sharper and less broadened than those obtained at lower temperatures. Reduced peak diffusion indicates improved mass-transfer kinetics and lower resistance to analyte transport within the porous stationary phase. This effect leads to enhanced separation efficiency and improved chromatographic resolution. In contrast, at lower temperatures, stronger analyte–polymer interactions and slower diffusion processes result in broader peaks and substantially prolonged retention times.

The obtained results confirm that thermally stable porous copolymers are particularly advantageous as stationary phases for high-temperature gas chromatography. Their combination of high thermal resistance, developed porous structure, and the presence of polar ester functionalities enables efficient separation of polar organic compounds within a relatively short analysis time while maintaining good peak shape and separation performance.

## 4. Conclusions

Porous poly(DMN-co-TRIM) microspheres based on the newly synthesized aromatic monomer 1,4-(dimethacryloyloxymethyl)naphthalene (DMN) and trimethylolpropane trimethacrylate (TRIM) were successfully prepared using the suspension–emulsion polymerization technique. The applied synthetic approach enabled the formation of spherical microspheres with a well-developed porous structure and good thermal stability. The obtained materials exhibited high specific surface areas reaching up to 494 m^2^/g, confirming the effectiveness of toluene as a porogenic diluent in the formation of porous crosslinked networks. The porous structure of the copolymers was strongly dependent on the monomer composition. Increasing the TRIM content resulted in higher crosslinking density, earlier phase separation during polymerization, and formation of a finer porous architecture characterized by higher specific surface area and lower pore diameters. The results demonstrated that the textural properties of the materials could be effectively controlled through adjustment of the DMN/TRIM ratio. Post-polymerization modification with tetrachloromethane in the presence of anhydrous AlCl_3_ caused moderate but composition-dependent changes in the porous structure. The highly crosslinked copolymers preserved their porous architecture to a large extent, indicating considerable structural resistance of the DMN–TRIM network toward Friedel–Crafts-type treatment. In comparison with previously investigated styrene–divinylbenzene systems, the poly(DMN-co-TRIM) materials exhibited greater dimensional stability and lower susceptibility to extensive pore reconstruction during modification. Thermogravimetric investigations confirmed the high thermal stability of both the parent and modified copolymers. All materials decomposed according to a two-step degradation mechanism typical of highly crosslinked methacrylate networks. Increasing the TRIM content systematically improved the resistance toward advanced thermal degradation, reflected by increasing T_50%_ and higher temperatures of the second DTG maximum. Chemical modification slightly decreased the temperature of the second degradation stage, most likely due to the introduction of thermally less stable chlorinated fragments, but simultaneously increased the amount of char residue, indicating altered degradation pathways and enhanced carbonization tendency. The synthesized copolymers also demonstrated promising properties as stationary phases for gas chromatography. Their high thermal resistance enabled operation at elevated temperatures, while the presence of ester groups within the polymer structure provided favorable interactions with polar analytes such as aliphatic alcohols. The chromatographic experiments confirmed efficient separation performance, particularly at higher operating temperatures, where shorter retention times and sharper chromatographic peaks were obtained.

Overall, the presented study demonstrates that porous poly(DMN-co-TRIM) microspheres constitute a new class of thermally stable aromatic methacrylate-based materials with a tunable porous structure. The results also demonstrate that the pore size distribution must be carefully optimized for the intended application. Although increasing the TRIM content resulted in higher specific surface areas accompanied by a shift toward smaller pore diameters, excessive pore narrowing may limit the accessibility of the internal surface and reduce the diffusion rate of larger molecules within the porous network. Therefore, the most suitable material should be selected by balancing surface area and pore dimensions according to the requirements of a particular application. In gas chromatographic separations and adsorption processes involving small molecules, narrower mesopores may enhance interactions with analytes, whereas applications involving larger molecular species require sufficient pore accessibility to ensure efficient mass transport.

## Figures and Tables

**Table 1:** Designations of copolymers and basic parameters of their porous structure.

Copolymeric Microsphere	Monomers Ratio	Specific Surface Area *S*_BET_ (m^2^/g)	Pore Volume *V* (cm^3^/g)	Pore Diameter *D*_BJH_ (nm)
poly(DMN-co-TRIM)_1	1:1	368	0.426	3.8/12 *
poly(DMN-co-TRIM)_2	1:2	386	0.381	3.5/7.5 *
poly(DMN-co-TRIM)_3	1:3	401	0.560	4/6 *
poly(DMN-co-TRIM)_4	1:4	480	0.442	3.9
poly(DMN-co-TRIM)_5	1:5	494	0.450	3.7

*—bimodal pore size distribution.

**Table 2:** Designations of the modified copolymers and basic parameters of their porous structure.

Copolymeric Microsphere	Monomers Ratio	Specific Surface Area *S*_BET_ (m^2^/g)	Pore Volume *V* (cm^3^/g)	Pore Diameter *D*_BJH_ (nm)
poly(DMN-co-TRIM)_1c	1:1	383	0.456	3.5/10 *
poly(DMN-co-TRIM)_2c	1:2	403	0.398	3/9 *
poly(DMN-co-TRIM)_3c	1:3	416	0.535	4/6
poly(DMN-co-TRIM)_4c	1:4	387	0.422	4
poly(DMN-co-TRIM)_5c	1:5	498	0.447	3.9

*—bimodal pore size distribution.

**Table 3:** The swellability coefficients of the parent and modified copolymers.

Copolymer	Swellability Coefficient *B* (%)			
	Methanol	Toluene	Acetone	1,2-Dichlorethane
poly(DMN-co-TRIM)_1	24	28	31	34
poly(DMN-co-TRIM)_2	26	29	32	37
poly(DMN-co-TRIM)_3	30	32	39	41
poly(DMN-co-TRIM)_4	25	30	32	33
poly(DMN-co-TRIM)_5	23	29	29	32
poly(DMN-co-TRIM)_1c	20	22	24	28
poly(DMN-co-TRIM)_2c	21	20	22	25
poly(DMN-co-TRIM)_3c	23	23	25	30
poly(DMN-co-TRIM)_4c	19	18	19	22
poly(DMN-co-TRIM)_5c	18	17	17	21

**Table 4:** Characteristic thermal degradation temperatures of the parent copolymers determined from TG and DTG analyses performed in helium.

Copolymeric Microspheres	*T*_5%_ (°C)	*T*_20%_ (°C)	*T*_50%_ (°C)	*T*_max1_ (°C)	_Δ_*m*_1_ (%)	*T*_max2_ (°C)	_Δ_*m*_2_ (%)	Residue (%)
**poly(DMN-co-TRIM)_1**	301	331	400	331	45.9	446	52.6	1.5
**poly(DMN-co-TRIM)_2**	300	333	406	333	39.9	445	57.2	2.9
**poly(DMN-co-TRIM)_3**	295	330	410	334	40	450	58	0.1
**poly(DMN-co-TRIM)_4**	292	333	401	331	40	453	59	0.9
**poly(DMN-co-TRIM)_5**	294	324	415	324	39.8	453	57.7	0.9

**Table 5:** Characteristic thermal degradation temperatures of the modified copolymers determined from TG and DTG analyses performed in helium.

Copolymeric Microspheres	*T*_5%_ (°C)	*T*_20%_ (°C)	*T*_50%_ (°C)	*T*_max1_ (°C)	_Δ_*m*_1_ (%)	*T*_max2_ (°C)	_Δ_*m*_2_ (%)	Residue (%)
**poly(DMN-co-TRIM)_1c**	301	328	386	330	44.5	417	45.5	10
**poly(DMN-co-TRIM)_2c**	293	336	405	323	33	416	51.5	15.5
**poly(DMN-co-TRIM)_3c**	292	334	405	327	44.2	418	48	7.8
**poly(DMN-co-TRIM)_4c**	306	340	404	331	38.5	433	54	7.5
**poly(DMN-co-TRIM)_5c**	298	335	405	333	35	424	57	7.3

## Data Availability

The original contributions presented in this study are included in the article. Further inquiries can be directed to the corresponding authors.
